# Partial monovision achieved by unilateral implantation of a multifocal add-on lens with bilateral pseudophakia: evaluation and results

**DOI:** 10.1007/s00417-022-05584-y

**Published:** 2022-02-17

**Authors:** Vitus André Knecht, Horaţiu Alexandru Colosi, Andrea Hassenstein

**Affiliations:** 1 Department of Ophthalmology, University Medical Center Hamburg-Eppendorf, Hamburg, Germany; 2 Department of Ophthalmology, Charité University Medicine Berlin, Mittelallee 4, Augustenburger Platz 1, 13353 Berlin, Germany; 3 Department of Medical Education, Division of Medical Informatics and Biostatistics, Iuliu Hatieganu University of Medicine and Pharmacy, Cluj-Napoca, Romania

**Keywords:** Partial monovision, Presbyopia correction, Cataract surgery, Refractive surgery, Multifocal AddOn®, Unilateral trifocal AddOn®

## Abstract

**Purpose:**

To assess the results of partial monovision (PMV) in comparison to a bilateral monofocal implantation (MMV).

**Methods:**

The PMV group was treated bilaterally with a monofocal intraocular lens (IOL) implantation, followed 3 months later by the implantation of a multifocal AddOn® lens (+ 3.00 D) into the non-dominant eye. The MMV group received a bilateral monofocal IOL implantation intending to achieve a slight anisometropia (0.0 D/ − 0.50 D). The near visual acuity (UNVA), intermediate visual acuity (UIVA), distance visual acuity (UDVA), defocus curve, and Lang-Stereotest II were conducted uncorrected, binocular, and minimum 3 months after the last operation. For the contrast sensitivity test, the patients were refractively corrected. The Quality of Vision Questionnaire (QoV), Visual Function Questionnaire (VF-14), spectacle independence, and general satisfaction were also assessed.

**Results:**

A total of 27 PMV patients and 28 MMV patients without ocular diseases relevant to visual acuity were examined. The PMV group was significantly better at UNVA (0.11 ± 0.08 logMAR vs 0.56 ± 0.16 logMAR) and between − 2.00 and − 4.00 D in the defocus curve (*p* < 0.001). At the UIVA, the PMV group was slightly better (0.11 ± 0.10 logMAR vs 0.20 ± 0.18 logMAR) but not significant (*p* = 0.054). The UDVA (− 0.13 ± 0.09 logMAR vs − 0.09 ± 0.14 logMAR) (*p* = 0.315) and contrast sensitivity (*p* = 0.667) revealed no differences between the groups. The stereo vision was in favor of PMV (*p* = 0.008). Spectacle independence was statistically better for PMV at distance, intermediate, and near (distance *p* = 0.012; intermediate *p* < 0.001; near *p* < 0.001). In the VF-14 Questionnaire, the PMV was statistically superior (*p* < 0.001). The QoV Questionnaire showed no differences regarding frequency and severity of visual disturbances. Both groups were highly satisfied (*p* = 0.509).

**Conclusion:**

Patients with PMV are more independent of glasses and are able to read without disadvantages in distance vision, due to halos and glare. The concept of PMV is well suited for the desire of eyeglass independence, without optical side effects.

**Supplementary Information:**

The online version contains supplementary material available at 10.1007/s00417-022-05584-y.


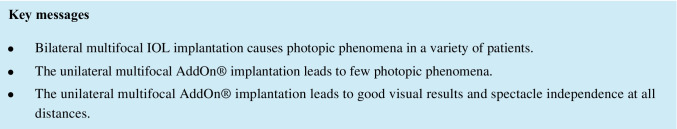


## Introduction

Presbyopia is an age-related decrease in lens elasticity with a progressive loss of accommodation. Patients can no longer see clearly in the near range and need reading glasses [1]. The correction of presbyopia is achieved with a near addition, which is up to 3 diopters (D). After a cataract operation with target refraction emmetropia, reading glasses or progressive glasses are required for the near and intermediate distance. However, many patients wish to live without glasses. Surgical correction by multifocal intraocular lenses (IOLs), monovision, and refractive corneal surgery has not yet led to an optimal solution [1]. Approximately 4% of patients in Europe choose a multifocal lens [2]. This reveals the doubts of doctors and patients about the available methods.

Patients with a multifocal IOL or a refractive corneal surgery feel disturbed by the decreased quality of far vision (waxy vision), the reduced reading quality especially in poor lighting, halos, glare in the dark, and deviation from the target refraction (blurred vision). In literature, it is therefore recommended that patients should be selected with caution, taking into account the personality and needs of the patient [3]. Trifocal lenses have largely replaced bifocal lenses, as they are clearly superior in the intermediate range [4]. In the last few years, enhanced depth of focus (EDOF) lenses with a single elongated focal point have entered the market. EDOF lenses achieve better results in the intermediate range, e.g., on the computer, and have fewer optical side effects. The disadvantage is the weakened near range compared to trifocal lenses [5]. To overcome the disadvantages of both lens systems, some surgeons combine them in a mix-and-match approach [6]. Furthermore, multifocal IOLs can be implanted in the sulcus in cases of pseudophakia. A better centration of the sulcus IOL was observed with the advantage of reversibility [7, 8].

In monovision, the dominant eye is set with a monofocal IOL for distance vision, while the non-dominant eye receives a monofocal IOL for near vision. The use of monovision is limited because the difference of 2 diopters between the two eyes can lead to binocular vision disorders and is then poorly tolerated by patients. The stereoscopic vision is less in monovision. Small differences in refraction are better tolerated, but do not allow reading without glasses [9].

The combination of both methods, monovision and the implantation of a multifocal lens in one eye, leads to a modified monovision: a partial monovision. In this procedure, one eye receives a monofocal IOL, and the other eye a multifocal system. Patients of the control group (MMV) were treated bilaterally with monofocal IOLs with the aim of achieving a slight anisometropia of half a diopter.

The selection of a monofocal comparison group was made to show that the PMV patients have a superior reading ability, without loss of far and contrast vision and their quality of vision due to glare and halos.

The aim of the study has been to investigate whether PMV can avoid the disadvantages of monovision and bilateral multifocal IOL implantation and can help patients become spectacle independent without side effects, by using various vision tests and subjective questionnaires.

## Methods

### Study setting and design

This monocentric observational retrospective cohort study was conducted unblinded and not randomized with the consent of the Ethics Commission Hamburg. The study was conducted as a pilot study planning to examine 30 patients per group [10]. The groups were matched for the overall proportion of age, gender, and type of surgery (clear lens extraction (CLE) or cataract). The surgeries were performed before the start of the study after detailed medical consultation and at the patients’ request. This allowed us to access 2 patient pools retrospectively. We recruited a total of 60 patients from these 2 patient pools consecutively.

Inclusion criteria for participation in the study were no previous eye or systemic diseases that could have a negative impact on vision and surgery without complications. The examination took place at the earliest 3 months after the last operation.

In addition to their study participation consent, the patients have agreed to the data protection concept and the anonymized use of their data. The IOL implantation was performed by the same surgeon who was not involved in the patient selection to avoid bias. The eyes were measured with the IOL Master 500 from Zeiss (Carl Zeiss AG). In addition, a measurement with the iTrace from Tracy (Tracey Technologies LLC) was performed to exclude higher-order aberrations, calculate toric lenses, and measure the angle kappa.

The partial monovision is performed in 3 steps: First, a monofocal IOL is implanted in the patient’s non-dominant eye. In the second step, which takes the refraction result of the first eye into account, a monofocal IOL is implanted into the dominant eye. In the third step, a multifocal AddOn® lens is implanted in the sulcus anterior to the monofocal IOL in the non-dominant eye after 3 months. This approach limits monovision to the near range.

The target refraction for the control group was 0 D in one eye and − 0.5 D in the other eye resulting in a mild monovision, as is often the practice for a monofocal lens implantation.

### Details of the lenses used

The monofocal models in the study were the aspherical, non-toric lenses: Hoya iSert® 250, 255, Vivinex iSert® XC1, XY1, and AcrySof® IQ. The AcrySof® IQ Toric IOL was used as the toric monofocal lens model.The selection of the monofocal lenses was made by the patients after personal considerations with the help of the surgeon. The multifocal add-on model was the 1stQ A4DW0M, a diffractive multifocal AddOn® lens with trifocal optic through elevated phase shift diffractive array—6 steps, apodized, the constructive interferences create an intermediate peak. The spherical and toric correction of residual refractive errors is possible.

The refractive power of the AddOn® IOL was calculated based on the refractive error (subjective refraction) of the eye. An online calculator from 1stQ is available for this purpose or the measured values are sent to the manufacturer who calculates the IOL for the patient.

### Study outcomes

Regarding the primary outcomes, the following tests were performed binocularly uncorrected under standardized conditions in a single session. First, the uncorrected distant visual acuity (UDVA) was measured at 6 m using a Möller-Wedel M 2000 projector (Möller-Wedel GmbH). After that, the uncorrected intermediate visual acuity (UIVA) was tested at a distance of 80 cm with Sloan letters in ETDRS format from Precision Vision (Precision Vision, Inc.). Uncorrected near visual acuity (UNVA) was documented with Precision Vision’s mixed Colenbrander contrast plate at 100% and 10% contrast. For reading ability, the last fluently presented sentence was evaluated. The uncorrected binocular defocus curve was performed with Möller-Wedel instruments, the M 2000 projector, and the Visutron Plus phoropter. The visual acuity was documented in 0.5 diopter steps from + 1 D on to − 4 D. The Lang-Stereotest II was applied. Only for the low-contrast distant visual acuity the patients were refractively corrected, in order not to influence the result by refractive errors. The contrast sensitivity was tested at 6 m with the computer program Freiburg Vision Test (“FrACT”) by Prof. Bach [11].

In addition to the objective eye tests, as secondary outcomes, the subjective satisfaction values were also surveyed. With the Quality of Vision Questionnaire (QoV) [12], patients were asked about various photopic phenomena and their frequency, severity, and impairment. The Visual Function Questionnaire (VF-14) [13] explores how patients can manage everyday situations, but in our study without wearing glasses.

Patients were inquired about their independence of glasses for the distance, the intermediate range, and the near range. Furthermore, the general satisfaction was assessed on a scale from 0 to 10 (0, totally dissatisfied/10, completely satisfied).

### Statistical analysis

Data were collected using Excel spreadsheets (Microsoft Corp.). Continuous variables with normal distribution have been compared using *t*-tests for independent samples. For continuous variables with a non-normal distribution, Mann–Whitney tests have been applied. Chi-square tests were used for the frequency analysis of qualitative variables. A Rasch analysis of the QoV was performed. For the estimation of the RASCH model, the Software Package R (version 4.0.2) was used. The function tam.mml() of the package TAM was applied for fitting the model, where a marginal maximum likelihood approach was chosen to determine the model coefficients. The answers to the QoV Questionnaire were ranked on a linear interval scale (0 to 100). Here, lower Rasch-weighted QoV scores indicate better vision quality. Differences between the compared groups which rendered *p* values lower than 0.05 were considered statistically significant. Analyses have been performed using IBM SPSS Statistics 25 (IBM Corp.).

## Results

### Demographics

In total, 27 PMV patients and 28 MMV patients were examined and evaluated. All 60 patients wanted to participate in the study, but during the 2 weeks of scheduled examinations, 5 patients could not attend due to illness, vacation, or job-related reasons.

The demography of the studied sample is presented in Table 1. No significant differences have been found between the groups concerning age, gender, type of surgery, follow-up, preoperative spherical equivalent (SEQ), and biometry. Postoperatively, the target refraction of emmetropia in PMV and mild anisometropia of half diopter myopia/emmetropia in MMV was achieved. This intended refractive difference was confirmed as statistically significant. Toric lenses were frequently used in both groups, PMV 56% and MMV 38% of the eyes (*p* = 0.057).

**Table 1:** Demography of the studied sample

	Mean ± SD range: (X to X) median: (X)		*p* value
	PMV	MMV	
*N* (patients)	27	28	/
Age (in years)	58.89 ± 8.96 (41 to 72)	57.29 ± 7.18 (45 to 69)	0.470
Gender (female/male)	13/14	16/12	0.504
Type of operation (CLE/cataract)	12/15	10/18	0.509
Follow-up (days)	421 ± 279 (106 to 1111)	335 ± 215 (99 to 969)	0.386
Preoperative SEQ (D)	− 1.24 ± 4.10 (− 12.56 to + 3,19)	− 1.41 ± 4.98 (− 11.94 to + 9.00)	0.669
AL (mm)	24.12 ± 1.40 (21.83 to 27.49)	23.10 ± 1.58 (20.79 to 28.36)	0.613
K (D)	43.80 ± 1.73 (39.22 to 47.14)	43.62 ± 1.81 (39.41 to 46.36)	0.888
CYL (D)	− 0.97 ± 0.85 (− 0.23 to − 3.57)	− 1.09 ± 0.89 (− 0.11 to − 3.47)	0.839
CYL axis (°)	104.43 ± 71.48 (143)	84.63 ± 68.59 (70)	0.092
ACD (mm)	3.25 ± 0.38 (3.29)	3.15 ± 0.38 (3.08)	0.222
IOL power (D)	19.14 ± 4.06 (10 to 25)	19.80 ± 5.15 (8 to 30)	0.303
Toric lens (%)	30/54 (56%)	21/56 (38%)	0.057
Postoperative SEQ (D)	− 0.06 ± 0.39 (0)	− 0.19 ± 0.43 (− 0.25)	0.049

*CLE* clear lens extraction, *SEQ* spherical equivalent, *AL* axial length, *K* keratometry equivalent, *CYL* corneal cylinder, *CYL axis* corneal cylinder axis, *ACD* anterior chamber depth, *IOL* intraocular lens, *SD* standard deviation, *PMV* partial monovision group, *MMV* monofocal group

### Visual acuity

Binocular UNVA showed significantly better results in all categories in favor of PMV (*p* = 0.001). The PMV group showed 100% of patients (pat.) with ≥ 0.30 logMAR and 96% ≥ 0.20 logMAR. In comparison, 14% of patients ≥ 0.30 logMAR and 0% ≥ 0.20 logMAR were found in the MMV group.

Also, the binocular UIVA at 80 cm showed better results of PMV, but not statistically significant (*p* = 0.054). PMV was better than MMV by 0.10 logMAR on average and median. The PMV group showed 100% of patients with ≥ 0.30 logMAR and 96% with ≥ 0.20. In the MMV group, the results were 75% of patients with ≥ 0.30 and 57% with ≥ 0.20. The PMV achieved a mean of − 0.13 logMAR in binocular UDVA versus MMV with − 0.09 logMAR. Statistical analysis showed no significant difference between the two groups (*p* = 0.613).

**Fig. 1:**
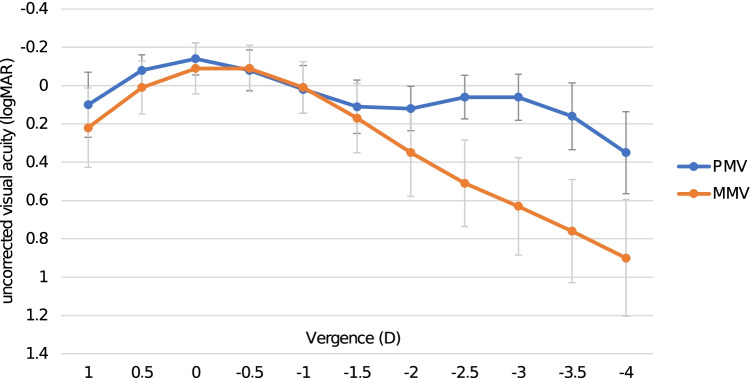
Uncorrected binocular defocus curve. Note: Vertical bars denote standard deviation. Abbreviations: PMV, partial monovision group; MMV, monofocal group; D, diopter; logMAR, logarithm of the minimum angle of resolution

The results for visual acuity from Table 2 also matched the uncorrected defocus curve in Fig. 1. Where MMV reached similar values as PMV, but from − 2 D to − 4 D, the defocus curve of MMV was significantly worse (*p* = 0.001). Over the whole length of the defocus curve, the PMV had a lower standard deviation (SD); only at − 1 D, the SD is the same.

**Table 2:** Visual acuity in logMAR

	Mean ± SD median: (X)		*p* value
	PMV	MMV	
UDVA (6 m)	− 0.13 ± 0.09 (− 0.20)	− 0.09 ± 0.14 (− 0.10)	0.315
UIVA (80 cm)	0.11 ± 0.10 (0.10)	0.20 ± 0.18 (0.20)	0.054
UNVA (40 cm)	0.11 ± 0.08 (0.10)	0.56 ± 0.16 (0.60)	< 0.001
UNVA reading ability (40 cm)	0.14 ± 0.10 (0.10)	0.54 ± 1.44 (0.60)	< 0.001
UNVA 10% contrast (40 cm)	0.39 ± 0.13 (0.40)	0.70 ± 0.13 (0.70)	< 0.001

*logMAR* logarithm of the minimum angle of resolution, *UDVA* uncorrected distance visual acuity, *UIVA* uncorrected intermediate visual acuity, *UNVA* uncorrected near visual acuity, *SD* standard deviation, *PMV* partial monovision group, *MMV* monofocal group

### Stereoacuity and contrast sensitivity

When comparing corrected contrast sensitivity, no significant differences were found in Table 3 (*p* = 0.667).

**Table 3:** Contrast sensitivity and stereo vision

	Mean ± SD median: (X)		*p* value
	PMV	MMV	
Contrast sensitivity (6 m)	1.77 ± 0.11 (1.81)	1.76 ± 0.22 (1.81)	0.667
	Number of patients: X/X (X%)		
Lang-Stereotest II uncorrected			0.008
P: positive D: doubtful N: negative	P: 19/27 (70%) D: 4/27 (15%) N: 4/27 (15%)	P: 8/28 (29%) D: 9/28 (32%) N: 11/28 (39%)	

*SD* standard deviation, *P* positive, *D* doubtful, *N* negative, *PMV* partial monovision group, *MMV* monofocal group

The uncorrected Lang-Stereotest II was positive in 70% of PMV patients and in 29% of MMV patients, resulting in a statistically significant difference in stereo vision (*p* = 0.008).

### VF-14 Questionnaire

The VF-14 Questionnaire, SI 1. VF-14, confirmed the results of the visual tests. There was a high statistical significance in favor of PMV in the questions referring to near range 1, 2, 7, and 8. There was no statistically significant advantage of the MMV group in any question. Without any exception, the self-perceived level of PMV was at a score of 4 (no difficulty at all), while MMV was between 0 (not possible) and 2 (some difficulties) regarding the questions about near range. Both groups had no difficulty driving at night without glasses.

### Spectacle independence

As listed in Table 4, PMV patients were more likely to be spectacle independent than MMV patients at near and intermediate distances. At distance, all PMV patients were spectacle free compared to 14% of patients in the MMV group who always wear spectacles. The general satisfaction was on average higher in the MMV group, while there was no difference between the groups regarding the self-perceived median score of 9 points.

**Table 4:** Spectacle independence

	Mean ± SD number of patients: X/X (X%)		*p* value
	PMV	MMV	
1.1 How often do you wear glasses for any purpose?	0.41 ± 0.50 (0) 0: 16/27 (59%) 1: 11/27 (41%) 2: 0/27 (0%)	1.00 ± 0.54 (1) 0: 4/28 (14%) 1: 20/28 (71%) 2: 4/28 (14%)	< 0.001
1.2 How often do you wear glasses for near tasks (e.g., reading print)?	0.48 ± 0.58 (0) 0: 15/27 (56%) 1: 11/27 (41%) 2: 1/27 (5%)	1.71 ± 0.66 (2) 0: 3/28 (11%) 1: 2/28 (7%) 2: 23/28 (82%)	< 0.001
1.2 How often do you wear glasses for intermediate tasks (e.g., computer)?	0.30 ± 0.72 (0) 0: 23/27 (85%) 1: 0/27 (0%) 2: 4/27 (15%)	1.32 ± 0.95 (2) 0: 9/28 (32%) 1: 1/28 (4%) 2: 18/28 (64%)	< 0.001
1.3 How often do you wear glasses for distance tasks (e.g., driving)?	0.00 ± 0.00 (0) 0: 27/27 (100%) 1: 0 2: 0	0.36 ± 0.73 (0) 0: 22/28 (79%) 1: 2/28 (7%) 2: 4/28 (14%)	0.012
*SD* standard deviation; 0 = never; 1 = sometimes; 2 = always			
	Mean ± SD. Median: (X)		*p* value
2. General satisfaction (scale 0–10)	8.67 ± 1.62 (9)	9.04 ± 1.04 (9)	0.509
General satisfaction: 0 = totally dissatisfied; 10 = completely satisfied *PMV* partial monovision group, *MMV* monofocal group			

### Quality of Vision Questionnaire

**Fig. 2:**
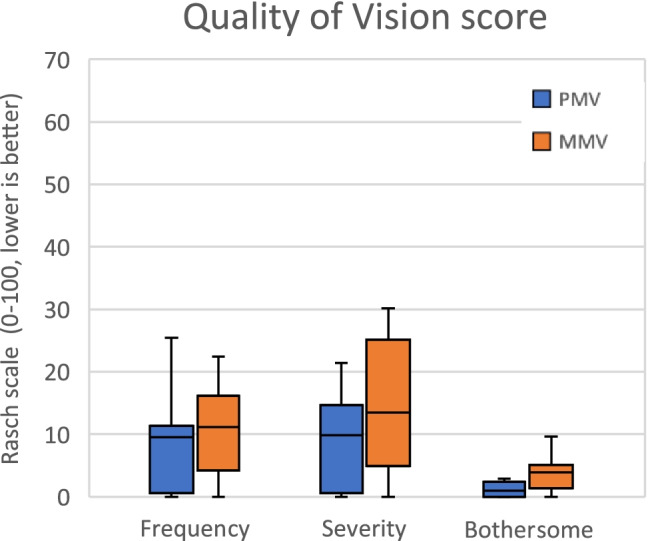
Quality of Vision score. Item summary of frequency, severity, and bothersome on the *x*-axis. Rasch scale (0–100) on the *y*-axis. Boxplot shows median; 25–75%; min–max. Abbreviations: PMV, partial monovision group; MMV, monofocal group

The results of the Quality of Vision Questionnaire are shown in Fig. 2, where low scores on the Rasch scale indicate fewer optical disturbances. It appears that the PMV group had a slightly better performance at the QoV than the MMV group, but there was no statistically significant difference in frequency (PMV 8.24 ± 8.07 (9.54) vs MMV 13.51 ± 13.31 (11.18) (mean ± SD (median)) (*p* = 0.288) and severity (PMV 8.87 ± 7.87 (9.84) vs MMV 15.27 ± 11.47 (13.48)) (*p* = 0.172) of visual disturbances. The difference in the degree of bothersome (PMV 1.20 ± 1.21 (1.02) vs MMV 3.85 ± 2.93 (3.89)) (*p* = 0.020) was significant. In total, 85% of the PMV patients did not experience any halos and no single PMV patient noticed halos “very often”. Then, 93% of the PMV patients were not bothered by halos at all; this accounts for 89% patients in the MMV group.

## Discussion

Our study has shown that PMV patients achieved good reading visual acuity with no disadvantages in distance vision. In addition, they were significantly more independent of glasses without increased perception of optical side effects such as halos and glare compared to the monofocal control group.

For more than 20 years, multifocal lenses have been used as an alternative to monofocal IOLs to achieve spectacle independence. Due to side effects, multifocal lenses could not be fully established until now. In Europe, about 4% of implanted lenses are multifocal lenses, as they can have disadvantages like reduced contrast sensitivity, halos, and glare [2]. There have been technical improvements and further developments in the field of multifocal IOLs over the years. Trifocal lenses have largely replaced bifocal lenses, being significantly better in the intermediate range [4].

In general, all these lenses reach the limits of physics and have specific side effects. The distribution of light to several focal points worsens contrast sensitivity and patients lose brilliance in distant vision [14].

Without exception, depending on the type of lens, about 15% of the light energy is lost due to the distribution of light [15]. As a result, patients complain about low-contrast vision (waxy vision) and difficulties reading in poorly illuminated conditions. In addition, patients are often disturbed by photopic phenomena such as halos and glare. This leads to considerable limitations in night vision and thus to numerous contraindications for implantation, such as professional driving [3].

As explained in the “Introduction”, enhanced depth of focus (EDOF) lenses with a single extended focal point have emerged in recent years, achieving better results in the intermediate range, and supposedly having fewer optical side effects. The disadvantage of trifocal IOLs in the intermediate range and EDOF IOLs in the near range is intended to be compensated with the mix-and-match approach [13]. A further problem when using multifocal lenses is the accuracy of the refractive result. In the everyday operative life, ophthalmic surgeons often only achieve a target accuracy of ± 0.5 D in 72.7% of cases. A target accuracy of ± 0.5 D and a cylinder of ≤ 1.0 D are only achieved in 55% of cases [16]. The goal of glasses-free vision often cannot be achieved without an additional correction with a Lasik touch-up. This Lasik touch-up frequently leads to an intensification of the already existing sicca syndrome and thus the optical side effects [17].

The treatment concept of PMV aims to avoid numerous problems of multifocal lenses. First, a monofocal IOL is implanted into the dominant eye, so that this eye should be free of optical phenomena such as halos and glare. The evaluations showed that the binocular perception of halos was not increased compared to bilateral monofocal implantation (*p* = 0.340). Compared to bilateral multifocal implantation, as, e.g., in the study by Monaco et al., halos were the most frequent phenomena (ca. 90% of patients) in both trifocal and EDOF groups; compared to their monofocal control group, the difference was significant [18]. The perception of halos in the PMV group (only 15% of patients) was substantially reduced relative to other studies [4, 19].

The monofocal IOL in the dominant eye is characterized by a higher optical quality compared to a multifocal IOL and therefore offers advantages for distance vision and contrast vision compared to bilateral trifocal lens implantation. The PMV showed a comparable good distance visual acuity to our bilateral MMV control group. The UDVA of PMV was higher compared to the trifocal studies of Cochener et al. and Mencucci et al. [20, 21]. In the study by Pilger et al., the monofocal group was statistically significantly superior to the EDOF group at UDVA [22].

The systematic three-step procedure increases the probability of an optimal refraction result. In order to achieve reliability, toric lenses were often used. The very good postoperative SEQ values and the low complaints about blurred vision in the PMV group support the three-step procedure. The studies of Gundersen et al. [23] and Gundersen and Potvin [24] have shown that ametropia is a major reason for postoperative correction in multifocal IOL implantation. In 8–12% of patients, a refractive correction is necessary. The implantation of a secondary lens (AddOn® lens) is a very good solution because sicca syndrome post-Lasik is avoided in elderly patients. Grundersen and Potvin [25] have examined the eyes for more than 3 years after AddOn® implantation and did not find intralenticular opacification and pigment dispersion. So far, none of the patients from PMV has been post-treated for a refractive error.

One might expect that the unilateral implantation of a multifocal IOL would lead to worse results in the intermediate and near range compared to the bilateral multifocal IOL implantation. However, the results of PMV obtained in UIVA are similarly good to those of bilaterally implanted EDOF and trifocal lenses reached in other studies [5, 20, 21]. A case series by Levinger showed that patients achieved very good UIVA and UNVA after unilateral refractive lens exchange with a trifocal lens in the non-dominant eye [26]. A retrospective observational case series by Fernández-García showed that bilateral refractive lens exchange with a trifocal lens was statistically better than unilateral one at UNVA with 0.04 ± 0.05 vs 0.09 ± 0.08 logMAR; no difference was found at UIVA [27].

In the current study, MMV also achieved reasonable results in the intermediary area, but the dependence on glasses of 64% of patients is higher than in the PMV group with 15%. PMV patients achieved uncorrected binocular visual acuity of at least 0.30 logMAR in 100% of the cases.

PMV also showed very good results in UNVA. In the near vision, we obtained a high degree of spectacle independence in 96% of PMV patients and achieved comparable good visual performance to bilateral trifocal IOLs [20, 21]. The results obtained seem to be better than those after bilateral EDOF implantation [5, 20, 21]. The MMV group with mild monovision was of course much weaker in the near range.

The binocular vision with a unilaterally implanted multifocal AddOn® lens was good in 70% of the cases and satisfactory in another 15% of the cases. Experiences of Kavassy [28] with the Lang test in adults with regular monocular and binocular functions indicated that optical fogging in one of the eyes would only disrupt the stereoscopic picture if visual acuity was less than 0.3. As expected, stereo vision in the MMV group without near correction was severely limited. In the study of Varón et al., bilateral multifocal implantation showed worse stereo acuity compared to bilateral monofocal implantation with near addition [29]. On the other hand, the study by Iida, Shimizu, and Ito concluded that the stereo acuity of unilateral multifocal implantation is significantly higher with a near addition [30].

The results achieved by PMV at the VF-14 were in every question at least equally good to the results obtained in the study by Alió, Vega-Estrada, and Plaza-Puche with bilateral multifocal lens implantation. Surprisingly, our patients rated their reading ability better than the patients in this study [31]. As we had hoped, the patients in our study had significantly less difficulty driving at night.

The achieved values of spectacle independence of PMV, as well as MMV, in the intermediate and near range, were comparable to the values achieved in the retrospective study by Rodov et al. investigating different lens systems [2].

Compared to bilateral monofocal implantation, PMV achieved equally good or better results at all distances. Compared to other studies with bilateral trifocal lens implantation, the visual results were better at distance and reached a similarly high level at intermediate and near distances [4, 5].

For years, literature has been advocating bilateral multifocal IOL implantation [32]. Our study showed that patients with unilateral implantation of a multifocal AddOn® lens did not have any problems with neuroadaptation. No patient has ever asked for the implantation of a second multifocal AddOn® lens or the explantation of one. The great flexibility of the central nervous system is supported by the fact that optical side effects are suppressed with this method.

The current study confirmed the expected advantages of the PMV treatment concept. This concept offers doctors and patients a wide range of options. After bilateral monofocal implantation, patients are offered an exit scenario. Patients can decide not to have a multifocal AddOn® lens implanted, which leads to an increased overall satisfaction of monofocal patients, which was also observed in our study. Cataract patients can only assess postoperatively with full visual acuity whether reading glasses are disturbing. After implantation of the multifocal AddOn® lens into the sulcus, an almost atraumatic explantation is possible at any time in case of intolerance or any occurrence of macular or retinal disease. The PMV also offers the option of extension, allowing a second multifocal AddOn® lens to be implanted in the partner eye in case of unsatisfactory intermediate visual acuity.

Our study is limited by the lack of a direct comparison to a bilateral trifocal or EDOF IOL implantation. Nevertheless, certain conclusions can be drawn from the comparison with the monofocal group as presented at the DOG 2020 [33]. A three-step approach with an additional surgery increases the risk of endophthalmitis and other surgical complications. The AddOn implantation is a short atraumatic procedure. The study by Shekhar et al. showed that the endophthalmitis rate was higher in resurgeries than in primary surgery. Especially eyes with a breach in the posterior capsule requiring vitrectomy and secondary IOL implantation had an increased risk of endophthalmitis (0.78%) vs no breach in the posterior capsule (0.02%) [34].

As a modification, it could be considered to implant the multifocal AddOn® lens during the surgery of the 1st eye. This is technically possible and could be performed on patients with assured accurate biometry and enhanced calculations. The advantages of the 3-step procedure, such as improved accuracy due correction with the AddOn® and the described exit scenario, would then have to be abandoned.

In our study, 17 AddOn® lenses without spherical correction and 10 AddOn® with max. ± 0.5 D were used. In one patient, the cylinder of ± 1 D was post-corrected with the AddOn®.

The method is designed to ensure that CLE patients, in particular, do not suffer a deterioration in the distance vision and optical quality compared to the status before surgery. The further brilliant distance visual acuity is essential for the satisfaction of this patient group.

In conclusion, it can be stated that patients with PMV were significantly more independent of glasses, have very good reading ability, no disadvantages in distance vision, and no optical side effects such as halos and glare.

The current study suggests that the PMV concept is well suited for the desire to be independent of glasses with only one multifocal add-on lens and without optical side effects.

## Supplementary Information

Below is the link to the electronic supplementary material.

Supplementary file1 (DOCX 22 KB)

## Data Availability

The datasets generated during and/or analyzed during the current study are available from the corresponding author on reasonable request.
