# Supplementary material for: Partial monovision achieved by unilateral implantation of a multifocal add-on lens with bilateral pseudophakia: evaluation and results
Source: Graefes Arch Clin Exp Ophthalmol. 2022 Feb 17;260(8):2753–62. doi: 10.1007/s00417-022-05584-y (PMC9325843; doi:10.1007/s00417-022-05584-y)
Supplement: Supplementary file 1 — Supplementary file1 (DOCX 22 KB) [file 417_2022_5584_MOESM1_ESM.docx]

| **SI 1. Visual Function Questionnaire (VF-14) without glasses** | | | |
| --- | --- | --- | --- |
|  | Mean±SD Median: (X) | |  |
|  | PMV | MMV | P - Value |
| 1. Do you have difficulty reading small letters like in a telephone book or in package inserts for  medications? (without glasses) | 3.48±0.80 (4) | 0.68±1.219 (0) | <0.001 |
| 2. Do you have difficulty reading a newspaper or a book?  (without glasses) | 3.70±0.542 (4) | 1.43±1.526 (1) | <0.001 |
| 3. Do you have difficulty reading big headlines and the  numbers on your telephone?  (without glasses) | 4.00±0.00 (4) | 3.43±0.96 (4) | <0.001 |
| 4. Do you have difficulty recognizing people who are  standing right in front of you?  (without glasses) | 4.00±0.00 (4) | 4.00±0.00 (4) | 1 |
| 5. Do you have difficulty (without glasses) noticing steps,  curbs, or stairs? | 4.00±0.00 (4) | 4.00±0.00 (4) | 1 |
| 6. Do you have difficulty (without glasses) noticing traffic,  information, or shop signs? | 3.89±0.42 (4) | 3.96±0.19 (4) | 0.521 |
| 7. Do you have difficulty (without glasses) sewing, knitting,  crocheting, or doing handicrafts? | 3.37±0.88 (4) | 1.86±1.69(1.50) | <0.001 |
| 8. Do you have difficulty (without glasses) filling out official  forms or cheques? | 3.78±0.506 (4) | 1.82±1.70 (2) | <0.001 |
| 9. Do you have difficulty (without glasses) playing cards,  chess, or dominos? | 4.00±0.00 (4) | 3.61±1.03 (4) | 0.043 |
| 10. Do you have difficulty (without glasses) performing sport  activities like tennis, golf, bowling, or boule? | 4.00±0.00 (4) | 4.00±0.00 (4) | 1 |
| 11. Do you have difficulty (without glasses) cooking? | 3.93±0.39 (4) | 3.93±0.38 (4) | 0.979 |
| 12. Do you have difficulty (without glasses) watching TV? | 4.00±0.00 (4) | 3.89±0.32 (4) | 0.083 |
| 13. How much difficulty do you have driving during the  daytime due to your vision problems?  (without glasses) | 4.00±0.00 (4) | 3.86±0.45 (4) | 0.083 |
| 14. How much difficulty do you have driving during the  night due to your vision problems?  (without glasses) | 3.63±0.49 (4) | 3.39±0.96 (4) | 0.692 |
| SD = standard deviation; 0 = not possible; 1 = a lot of difficulty; 2 =some difficulty; 3 = a little difficulty; 4 =no difficulty at all PMV= Partial Monovision group; MMV= monofocal group | | | |
